# Age-Related Deficits in Binocular Vision Are Associated With Poorer Inhibitory Control in Healthy Older Adults

**DOI:** 10.3389/fnins.2020.605267

**Published:** 2020-11-25

**Authors:** Grace Lin, Raghda Al Ani, Ewa Niechwiej-Szwedo

**Affiliations:** Department of Kinesiology, University of Waterloo, Waterloo, ON, Canada

**Keywords:** aging, stereopsis, executive functions, eye movements, antisaccade error rate

## Abstract

A robust association between reduced visual acuity and cognitive function in older adults has been revealed in large population studies. The aim of this work was to assess the relation between stereoacuity, a key aspect of binocular vision, and inhibitory control, an important component of executive functions. Inhibition was tested using the antisaccade task in older adults with normal or reduced stereopsis (study 1), and in young adults with transiently reduced stereopsis (study 2). Older adults with reduced stereopsis made significantly more errors on the antisaccade task in comparison to those with normal stereopsis. Specifically, there was a significant correlation between stereoacuity and antisaccade errors (*r* = 0.27, *p* = 0.019). In contrast, there were no significant differences in antisaccade errors between the normal and reduced stereopsis conditions in the young group. Altogether, results suggest that the association between poorer stereopsis and lower inhibitory control in older adults might arise due to central nervous system impairment that affects the processing of binocular disparity and antisaccades. These results add to a growing body of literature, which highlights the interdependence of sensory and cognitive decline in older adults.

## Introduction

Aging is associated with reduced physical strength, visual and hearing impairments, poorer memory, and lower scores on cognitive tests. These age-related changes were first documented in separate studies (Thompson, 2009; Owsley, 2011; Whitson et al., 2018); however, accumulating research has revealed a robust relation between visual and hearing deficits and lower performance on cognitive tests (Anstey et al., 2001b; Lin et al., 2004; Tay et al., 2006; Ong et al., 2012; Spierer et al., 2016; Chen et al., 2017; Ward et al., 2018). Previous studies that found a correlation between visual function and cognition focused on testing acuity and contrast sensitivity. Stereoacuity is an important aspect of binocular visual function that is often reduced in older adults (Wright and Wormald, 1992; Leat et al., 2013). However, the association between stereoacuity and cognitive function in older individuals has not been assessed to date. Therefore, the purpose of this study was to examine the relation between stereoacuity and one aspect of executive functions, namely, inhibitory control.

Evidence from large cross-sectional and longitudinal studies indicates that aging is associated with an increase in covariation between visual impairment and reduced cognitive function (Li and Lindenberger, 2002; Zheng et al., 2018). Specifically, the relation between vision and cognition is not significant in young or middle-aged adults, but it emerges in older adults (Baltes and Lindenberger, 1997). The association was first revealed in a large cohort of individuals aged 70–103 years, where visual acuity explained 41% of variance in intellectual functioning (Lindenberger and Baltes, 1994). These findings were subsequently confirmed and extended by the Australian longitudinal study of aging (ALSA), which included a cohort of 894 adults (70–98 years old) (Anstey et al., 2001a). Results showed that 78% of the age-related variance in cognition was shared with vision and hearing function. A robust relation between sensory and cognitive function persisted even when the cognitive assessment was performed using a test designed for people with low vision (Spierer et al., 2016). Importantly, inducing poor visual acuity experimentally in middle-aged adults was not associated with reduced performance on cognitive tests (Lindenberger et al., 2001). Overall, the research supports a robust correlation between visual impairment and poorer cognitive function in older individuals. Acuity has been the most commonly used measure to assess vision, and contrast sensitivity was used in one study (Ward et al., 2018). To date, no studies have examined the association between other aspects of visual function, such as binocular vision, and cognitive performance in older adults.

Binocular vision requires the ability to process inputs from both eyes. The two components of binocular function are stereopsis and ocular vergence, which are elicited by binocular disparity and provide relative and absolute depth cues (Howard and Rogers, 2002). Binocular viewing is associated with improved performance on many everyday tasks (Jones and Lee, 1981; Melmoth and Grant, 2006; Blake and Wilson, 2011). Processing of binocular disparity involves a distributed cortical network lateralized to the right, including the occipital, temporal, and partial areas (Nishida et al., 2001; Fortin et al., 2002; Koh et al., 2008), as well as the prefrontal cortex (Gulyas and Roland, 1994). Aging is associated with reduced binocular visual function (Leat et al., 2013). Notably, stereoacuity deteriorates more with age compared to high contrast visual acuity (Haegerstrom-Portnoy, 2005), and fewer than 30% of individuals 70–79 years old have normal stereoacuity thresholds (Zaroff et al., 2003). Age-related reduction in stereoacuity could arise due to changes in ocular alignment (i.e., phoria or tropia due to extraocular muscle weakness), reduced acuity in one or both eyes from cataracts or age-related macular degeneration, or changes in the central nervous system pathways involved in the processing of binocular disparity. In addition, studies have shown that individuals with neurodegenerative disorders, such as Alzheimer’s disease (Mittenberg et al., 1994), Parkinson’s disease (Koh et al., 2013), or glaucoma (Gupta et al., 2006), tend to have lower stereopsis. Although the association between binocular vision and cognitive function has not been examined in older persons without a diagnosed neurological disorder, a recent study revealed that poorer binocular function due to convergence insufficiency was correlated with lower inhibitory control in a cohort of young adults (Daniel and Kapoula, 2016).

Inhibition is an important aspect of executive functions, which is mediated by the prefrontal cortex (Langenecker et al., 2004; Tiego et al., 2018). The antisaccade oculomotor test has been used extensively to assess inhibitory control (Munoz and Everling, 2004). When performing an antisaccade, participants are instructed to look away from a target presented in the periphery, which requires inhibiting a reflexive saccade and remapping the saccade-related neural activity to the contralateral hemisphere. Importantly, this oculomotor test allows inhibition to be measured without relying on other visual (i.e., color vision, acuity) or cognitive (i.e., reading) processes, which can limit performance when using other clinical tests to evaluate inhibitory control (van Boxtel et al., 2001).

Inhibition of reflexive eye movements relies on an extensive neural network, which includes the frontoparietal areas, such as the dorsal lateral prefrontal cortex and the supplementary frontal eye fields, as well as the parietal eye fields (Ford et al., 2005; Brown et al., 2007). Research shows that inhibitory control, measured by the number of directional errors where participants fail to inhibit a reflexive saccade and look toward the target, matures over the first two decades of life, and the frequency of errors begins to rise again slowly in healthy middle-aged adults between 30 and 40 years old (Coe and Munoz, 2017). Extensive research reveals that aging is associated with a greater frequency of directional errors and increased latency of correct antisaccades (Olincy et al., 1997; Munoz et al., 1998; Butler et al., 1999; Peltsch et al., 2011; Noiret et al., 2017; Fernandez-Ruiz et al., 2018), suggesting that inhibitory control declines progressively with age in healthy older adults. Neuroimaging studies indicate that these age-related behavioral changes are correlated with a shift of neural activation from posterior to frontal areas, such that activity in visual and parietal areas is reduced while frontal activation is increased (Raemaekers et al., 2006; Alichniewicz et al., 2013).

To summarize, inhibitory control is a key component of executive functions, therefore, it is important to elucidate a better understanding of the influential individual variables associated with lower inhibition, particularly in older individuals. At present, a relationship between one aspect of binocular function (i.e., convergence insufficiency) and inhibitory control has been revealed in young adults (Daniel and Kapoula, 2016). However, little is known about the association between binocular visual function and inhibition in older adults. Therefore, the current study consisted of two experiments which sought to assess the relation between inhibitory control and one aspect of binocular vision, namely stereoacuity. Older adults with normal or reduced stereopsis were tested in experiment 1. It was hypothesized that older individuals with poorer stereopsis will have a greater number of errors on the antisaccade task and a longer latency for correct antisaccades. A second experiment was conducted with young adults with normal binocular vision to assess whether experimentally induced transient reduction of stereopsis affects inhibitory control. Results from this experiment provide insight into the mechanism underlying the correlation between reduced stereopsis and inhibition. Specifically, if transiently induced poor stereopsis is not associated with inhibitory control in younger adults, then the effects seen in older adults are more likely due to central nervous system changes affecting both binocular processing and inhibition rather than just a peripheral reduction in stereoacuity.

## Materials and Methods

### Experiment 1

#### Participants

A sample of convenience consisting of 94 community dwelling older adults (>66 years old) were recruited from the University of Waterloo Research and Aging Pool database. Nine participants were excluded from the study because of visual impairment due to age-related macular degeneration, glaucoma, or a scheduled cataract surgery. The Montreal Cognitive Assessment (MoCA) was used to assess general cognitive function and to ensure that participants scored within the normal range, which was considered as a score of 26 or greater (Nasreddine et al., 2005). Ten participants scored below 26 and were therefore excluded from the study. The final sample included 75 participants (*M* = 75.45 years of age, *SD* = 5.71): 59 females (age: 74.37 ± 5.19 years; education: 15.59 ± 2.79 years) and 16 males (age: 79.44 SD 5.97 years; education: 16.44 ± 3.41 years). The mean number of years of education was 15.77 (SD 2.92; range 12–26 years).

#### Procedure

The study protocol received ethics clearance through the Research Ethics Committee in accordance with the Declaration of Helsinki. All participants provided written consent. Testing was performed in a quiet, well-lit room. The study protocol included assessment of visual acuity and stereoacuity, which were performed under standard illumination. Participants wore their prescription spectacles during the assessment. Binocular and monocular visual acuities were tested at a 6 m distance using the Bailey-Lovie acuity chart. Visual acuity was defined as the lowest line for which at least three of the five optotypes were reported correctly. Stereoacuity was examined using the Randot Circles Stereotest (Stereo Optical Co. Inc.), which was administered according to the manual. The stereoacuity threshold was determined as the smallest disparity that was reported correctly. Participants were placed into one of two groups based on the results of the Randot test: stereo normal (stereoacuity threshold of 50 arc sec or better) or stereo reduced (stereoacuity threshold worse than 50 arc sec). Demographics and results from the vision tests for the two groups are shown in Table 1. A Wilcoxon two-sample test was performed to assess differences between groups. A significant difference was revealed between the groups, indicating that the stereo reduced group was on average older by 2.6 years.

**TABLE 1:** Demographics and visual function characteristics (mean ± SD) for participants with normal stereoacuity and reduced stereoacuity.

	**Normal stereoacuity (*n* = 39; 31 females) (threshold ≤ 50 arc sec)**	**Reduced stereoacuity (*n* = 36; 28 females) (threshold > 50 arc sec)**	**Wilcoxon test; *p*-value**
Age (years)	74.205.57	76.805.64	*Z* = 2.13; *p* = 0.033
Stereoacuity (arc sec)	35.7611.39	*182.22205.80	*Z* = 7.49; *p* < 0.001
Best corrected binocular visual acuity (logMAR)	0.050.10	0.080.09	*Z* = 1.19; *p* = 0.231
Interocular acuity difference (logMAR)	0.070.06	0.140.19	*Z* = 0.72; *p* = 0.470

*Participants with no measurable stereopsis were assigned a score of 800 arc sec.

Next, participants completed two oculomotor tasks: prosaccades and antisaccades, which were performed in separate blocks. The task and stimuli were designed following the recommendation outlined by Antoniades et al. (2013). Participants were seated at a distance of 80 cm away from a computer monitor (19 inch ViewSonic CRT monitor; resolution of 1,024 × 768 pixels; refresh rate of 85 Hz) with their chin placed in a chinrest. A video-based eye-tracker (EyeLink II; SR Research, Ontario, Canada) was used to record eye position at a sampling frequency of 250 Hz. The eye-tracker was calibrated using a 5-point calibration method. Validation of eye tracking was performed after the calibration, where the validation acceptance criterion was set at <1° error to ensure reliability.

The experimental protocol was created using the Experiment Builder software (ver. 1.8; SR Research, Ontario, Canada). At the initiation of each trial, participants focused their gaze on a black fixation cross (stimulus size 0.25°) shown at the midline at eye level and presented for a duration ranging between 1,500 and 2,250 ms. When the cross disappeared, a peripheral target (stimulus size 0.25°) was shown (step paradigm) at ±10° to the left or right of the fixation for 2,000 ms. In the prosaccade experimental condition, participants were instructed to look at the stimulus as quickly as possible. Participants completed one block of 50 trials in the prosaccade task. There was one block of antisaccade trials where participants were asked to look away from the target. Instructions were provided verbally, as well as on the computer monitor at the beginning of the pro and antisaccade block of trials. Directional errors were expected in the antisaccade task; therefore, 60 trials were conducted. The oculomotor tests were completed in 15 min.

#### Data Analysis

Eye movement data were analyzed offline using the eyetracker’s Data Viewer software (ver 1.8; SR Research, Ontario, Canada). Eye position data from each trial for both eyes were plotted and visually inspected. The eye which provided less noisy tracking results was selected for analysis. Trials were excluded if a blink or loss of eye tracking occurred within 100 ms prior to or 500 ms following target presentation. These criteria resulted in rejection of 8.5% of trials in the prosaccade task, and 8.6% of trials in the antisaccade task. Saccades were detected using the algorithm implemented in Data Viewer: 30°/s velocity threshold, and 8,000°/s^2^ acceleration threshold. The main outcome measure for the prosaccade task was saccade latency, which reflects speed of processing. For the antisaccade task, the main measures were directional error, which indicates difficulty in inhibiting a reflexive response, and latency of saccades made in the correct direction (i.e., away from the target).

Analysis of covariance (ANCOVA) was used to test for differences in inhibitory control between the groups (stereo normal and stereo reduced). Age was entered as a covariate because the stereo normal group was on average 2.6 years younger compared to the stereo reduced group. Although the interocular difference (IOD) was not significantly different between the groups, IOD was also entered as a covariate because larger IOD has been associated with reduced stereoacuity in previous studies (Lam et al., 1996). Statistical analyses were conducted using the Statistical Analysis System (SAS) Studio, ver. 3.5 Enterprise Edition (SAS Institute Inc., Cary, NC, United States).

### Experiment 2

#### Participants

Twelve healthy young adults were recruited (8 females; age: 24.1 years *SD* = 6.8). All participants provided written consent prior to testing. Participants had normal or corrected to normal visual acuity (≤0.0 logMAR) and stereoacuity (<50 arc sec).

#### Procedure

The same testing protocol was used as described in experiment 1. Participants completed one block of the prosaccade task and two blocks of the antisaccade task (one block with normal stereopsis, and one block with reduced stereopsis), which were randomized between participants. One of the antisaccade blocks was performed under normal viewing condition. Stereoacuity was reduced experimentally in the other antisaccade block using a convex lens, which was placed in front of the left eye. This manipulation reduces stereopsis because the retinal images are discordant (Melmoth et al., 2007; Niechwiej-Szwedo et al., 2012). The lenses used in the current experiment ranged between 2.25 and 3.50 prism diopters. Each participant was assessed prior to the experiment to determine which lens power should be used to increase the individual’s stereoacuity threshold to 200 arc sec.

## Results

### Experiment 1

Average binocular visual acuity, IOD, and stereoacuity thresholds for stereo normal and stereo reduced groups are reported in Table 1. Results from the Wilcoxon two sample test showed that acuity and IOD were not significantly different between the two groups. Average stereoacuity in the stereo normal group was 35.76 arc sec (*SD* = 11.39, *Mdn* = 30, *range* = 20–50). Three participants in the stereo reduced group had no measurable stereopsis, and the average stereoacuity for the remaining participants with measurable stereopsis was 126.06 arc sec (*SD* = 85.40, *Mdn* = 100, *range* = 70–400).

Results from a univariate analysis for the full cohort showed that mean prosaccade latency was 237 ms (*SD* = 43), which was in the range of slow regular latency saccades (i.e., 181–400 ms). Short latency saccades in the range of express saccades (i.e., 80–139 ms) were found on 3.7% of prosaccade trials, and 1.5% of antisaccade trials. Average antisaccade latency on correct trials was 370 ms (*SD* = 85). Overall, directional errors were found on 31% of antisaccade trials (*SD* = 18), and the average error latency on antisaccade trials was 249 ms (*SD* = 67), which was comparable to prosaccade latency.

**FIGURE 1:**
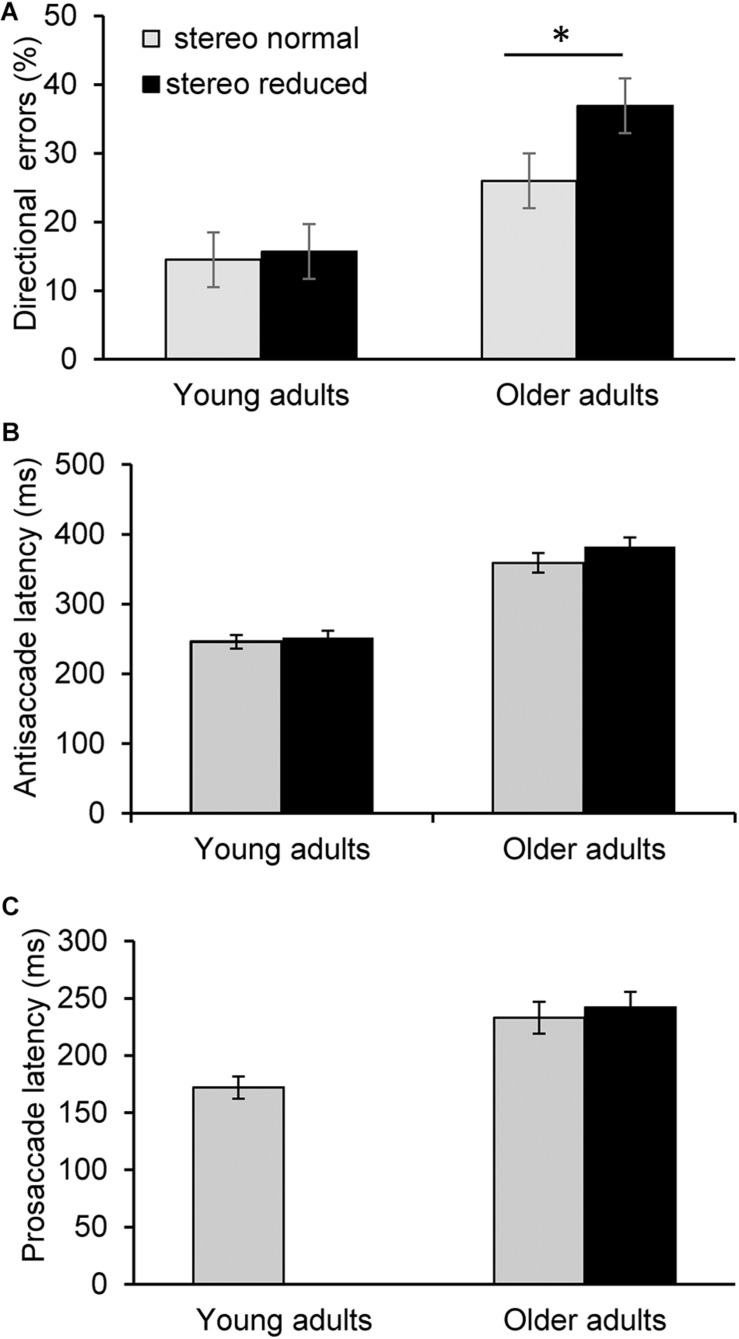
Saccade latency and error measures across task conditions. Error bars represent ± 1 standard error. Average percent of directional errors in the antisaccade task in young and older adults as a function to stereoacuity **(A)**. Older adults with reduced stereopsis exhibited significantly more errors (*p* < 0.05). In contrast, there was no significant difference in antisaccade errors between the stereo normal condition and when stereopsis was transiently reduced in young adults. There was no significant difference in antisaccade latency **(B)** or prosaccade latency **(C)** between the stereo conditions in the older or younger group. Prosaccade latency was not assessed as a function of reduced stereoacuity in the younger adults in Experiment 2. ^∗^*p* < 0.05.

**FIGURE 2:**
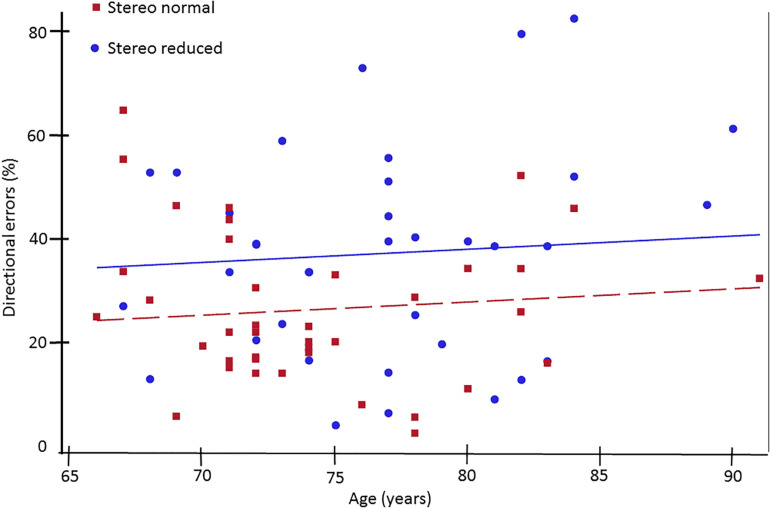
Percent of directional errors in the antisaccade task plotted as a function of age for older adults with normal (squares) and reduced (circles) stereoacuity. Individuals with normal stereoacuity made significantly fewer directional errors compared to individuals with reduced stereoacuity (*p* < 0.05).

Next, analysis was conducted to assess the influence of stereopsis on inhibitory control. Results from the ANCOVA adjusted for age and IOD revealed a significantly greater percent of directional errors in the antisaccade task in the stereo reduced group (36.92%, *SD* = 20.24) compared to the stereo normal group [25.99%, *SD* = 14.67; *F*_(1, 71)_ = 5.20, *p* = 0.026; Figure 1A]. Figure 2 shows the percentage of errors plotted for individuals in each group as a function of age, illustrating that individuals with reduced stereopsis were more likely to have more errors on the antisaccade task regardless of age. Additional Spearman correlation analysis was conduced to assess the strength of association between stereopsis and antisaccade error. Results showed a moderate correlation, *r*(74) = 0.27, *p* = 0.019, 95% CI [0.05–0.47]. The magnitude of correlation did not change after adjusting for age and IOD, *r*(71) = 0.27, *p* = 0.018.

There were no statistically significant differences between the groups with normal and reduced stereopsis for antisaccade latency [*F*_(1, 71)_ = 0.88, *p* = 0.350; Figure 1B] or prosaccade latency [*F*_(1, 71)_ = 0.11, *p* = 0.745; Figure 1C]. Average antisaccade latency was 359 ms (*SD* = 87) for the stereo normal group and 382 ms (*SD* = 82) for the stereo reduced group. Average prosaccade latency was 234 ms (*SD* = 34) for the stereo normal group and 241 ms (*SD* = 50) for the stereo reduced group. Additional analysis was conducted to assess whether the frequency of short latency (i.e., express) saccades was different between the groups with normal and reduced stereopsis. Express saccades occurred on 148 trials in the prosaccade task, with the majority (66%) occurring in the stereo normal group. Similarly, express saccades occurred on 74 trials in the antisaccades task with the majority (69%) occurring in the stereo normal group.

### Experiment 2

The aim of this experiment was to examine the effect of transiently reduced stereopsis on inhibitory control. As expected, the average latency of prosaccades was shorter (172 ms, *SD* = 24) in comparison to antisaccades, and there was no significant difference [*t*_(__11__)_ = -1.03; *p* = 0.325] between the two antisaccade conditions (stereo normal: 246 ms, *SD* = 34; stereo reduced: 252 ms, *SD* = 34). There was also no significant difference in the percentage of directional errors between the conditions where stereoacuity was normal (14.54%, *SD* = 16.20 and transiently reduced (15.69%, *SD* = 16.11; *t*_(__11__)_ = -0.54; *p* = 0.601; Figure 1A).

## Discussion

The primary goal of the current study was to assess the association between stereoacuity and inhibitory control in a cohort of healthy, community-dwelling older adults. A secondary goal was to determine if a transient reduction in stereoacuity affects inhibitory control in young adults. Results showed that older adults with reduced stereopsis exhibited a greater percentage of directional errors on the antisaccade task, which indicates poorer inhibitory control. This is the first study to report such an association, which extends the current knowledge about the interdependence of visual and executive functions in older individuals. Importantly, such correlation was not found when younger adults with experimentally induced poor stereopsis performed the antisaccade task. Therefore, it is unlikely that stereopsis is directly influencing inhibitory control. Instead, the association revealed in the current study could be indicative of a common age-related disruption in neural function that affects both the processing of binocular disparity and other aspects of executive function, specifically, inhibition.

A robust relation between sensory impairments and cognition has been reported in older individuals in large population studies (Anstey et al., 2001b; Li and Lindenberger, 2002; Ong et al., 2012; Zheng et al., 2018). Binocular visual acuity has been the most frequently used measure in previous studies, and the association with cognition was shown for both distance and near acuity. Because the current study sought to assess the relation between stereopsis and inhibition, only participants with relatively good binocular visual acuity, which was at least 0.3 logMAR (i.e., Snellen 20/40), were included. Consistent with previous studies (Zaroff et al., 2003; Haegerstrom-Portnoy, 2005), stereoacuity was significantly reduced in 48% of our cohort. As hypothesized, results revealed an association between stereopsis and inhibitory control, manifested as increased number of errors on the antisaccade task, which remained significant after controlling for age and interocular acuity difference. This indicates that individuals with poorer stereoacuity experienced greater difficulty inhibiting reflexive eye movements. In contrast, the latency of the correct antisaccades was not different between the groups with normal or reduced stereoacuity. Thus, antisaccade errors rather than latency appear to be a more sensitive marker of executive dysfunction. These findings are consistent with the results from studies conducted with adults with a history of concussion, which demonstrated that brain injury is associated with persistent errors on the antisaccade task even in cases when antisaccade latency is normal (Mani et al., 2018). The fact that stereopsis was correlated with inhibitory control in individuals with relatively good visual acuity suggests that stereoacuity may be a more sensitive test than acuity that could help to identify individuals with lower executive function. The cohort tested in the current study consisted of individuals with normal cognitive function, according to the MoCA test. However, it is possible that some individuals with poorer stereopsis might be at a greater risk of developing cognitive deficits, and this remains to be tested in longitudinal studies.

Stereopsis is one of the key measures of binocular vision and requires the ability to process inputs from both eyes. Stereoacuity deficits may arise due to optical factors that reduce acuity and contrast sensitivity (e.g., cataracts), photoreceptor degeneration, ocular muscle weakness that affects vergence control, or neural processing of binocular disparity. The results from our second experiment showed that a transient reduction in stereopsis is not associated with antisaccade errors, which suggests that optical image blur is not sufficient to impair inhibitory control. Thus, it is more likely that the deficits in stereopsis and inhibition in older adults arise due to a disruption in central nervous system processing that affects both of these functions.

Stereoscopic processing and the antisaccade task engage distributed neural networks, including extrastriate visual, parietal and prefrontal areas (Ford et al., 2005; Brown et al., 2007). Aging is associated with structural and functional changes in cortical networks, including reduced grey matter volume, white matter atrophy (Fjell et al., 2009; Liu et al., 2017), and compensatory activation patterns (Fernandez-Ruiz et al., 2018). Recent studies also highlight the relation between disruption in white matter tracts and age-related decline in sensorimotor and cognitive processing (Fling et al., 2011; Kohama et al., 2012). For example, microstructural changes in the corpus callosum have been found in apparently healthy older adults (Fan et al., 2019), and these changes have been linked with lower performance on various cognitive tasks (Sullivan et al., 2010; Wong et al., 2017; Halliday et al., 2019). It is possible that widespread disruption of white matter, including the corpus callosum, could lead to both reduced stereopsis and inhibitory processing because efficient performance of both tasks relies on activation of distributed cortical networks within and between the hemispheres. Specifically, processing of crossed disparities presented at midline stimulates the temporal part of each retina, and these inputs are first processed in different hemispheres. Thus, the splenium of the corpus callosum is involved in stereoscopic processing of small stimuli with crossed disparities presented at midline (Bridge, 2016). On the other hand, neuroimaging studies show that the antisaccade task activates an extensive frontoparietal network (Munoz and Everling, 2004; Ford et al., 2005), and improved selection and inhibition performance is associated with white matter integrity between these regions (Thakkar et al., 2016; Jarvstad and Gilchrist, 2019). Interestingly, fractional anisotropy, a measure of white matter integrity was correlated positively with saccade latency and lower behavioral cost of inhibition (Jarvstad and Gilchrist, 2019). Thus, lower white matter integrity may be contributing to poorer inhibition in our study. To summarize, deficits in stereoscopic processing and lower inhibitory control on the antisaccade task may arise due to a widespread age-related changes in white matter connectivity; however, imaging studies are needed to test this hypothesis directly.

The underlying mechanism to explain the relation between vision and cognitive function remains to be established. However, several hypotheses have been proposed (reviwed in: Li and Lindenberger, 2002). First, the sensory loss consequence hypothesis suggests that visual impairment leads to fewer social interactions and less cognitive stimulation, which is a risk factor for cognitive decline. Second, the resource allocation hypothesis speculates that individuals with sensory impairments require more neural resources to detect and discriminate sensory inputs, which leaves limited neural resources for cognitive processing. These two hypotheses postulate a causal relationship where deteriorating sensory function eventually leads to cognitive decline. Finally, the third hypothesis proposes a common cause for sensory and cognitive impairment. Although the current study cannot differentiate between these hypotheses, our results and interpretation are more consistent with the third hypothesis. Specifically, our results showed an association between a visual test (i.e., stereopsis) and a measure of executive function in the older cohort, but given the results of the second experiment, it is unlikely that reduced stereopsis directly leads to antisaccade errors. Instead, it is more likely that age-related microstructural and functional changes of the central nervous system could lead to a decrease in both sensory and cognitive function.

Our results provide new insight into the association between visual and cognitive function; however, it is important to acknowledge some limitations. First, the recruited participants included mainly women, while men represented less than a third of the cohort. Therefore, caution should be exercised when generalizing the results. Second, stereoacuity was assessed using a single clinical test which measures local stereopsis. Different clinical tests can be used to measure other components of stereopsis, such as global stereopsis. Research shows that local and global stereopsis rely on different neural networks (Ptito et al., 1991); thus, assessment of these different components of stereopsis could provide additional insight into the association with cognitive function in older individuals. Finally, individuals diagnosed with macular degeneration, glaucoma, or scheduled cataract surgery were excluded from the current study. However, these ocular disorders progress slowly, and it is possible that our cohort included individuals who had reduced stereopsis resulting from these conditions but were not yet formally diagnosed. In order to gain a fuller understanding into the relation between stereoacuity and cognitive function, future studies should include a more comprehensive assessment of visual and oculomotor function in order to stratify participants according to the underlying cause of stereopsis impairment.

In conclusion, this is the first study to reveal that reduced stereoacuity is correlated with poorer inhibitory control. This exploratory study cannot definitively reveal the nature of this association; however, it is possible that the deficits in processing of binocular disparity and inhibitory control are both due to a common impairment of neural function. Results from the current study have implications for future research investigating the interdependence of vision and cognitive function. Specifically, acuity has been the most commonly used measure of visual function in large population studies examining its relation to cognition. However, future studies should consider adding a stereoacuity assessment, which seems to be more closely associated with executive dysfunction. The findings from the current study improve our understanding of the nature of the interaction between sensory and executive functions, and this knowledge could be used to develop better diagnostic and prognostic tools to monitor older individuals at risk of cognitive decline.

## Data Availability Statement

The raw data supporting the conclusions of this article will be made available by the authors, without undue reservation, to any qualified researcher.

## Ethics Statement

The studies involving human participants were reviewed and approved by the University of Waterloo Ethics Board. The patients/participants provided their written informed consent to participate in this study.

## Author Contributions

GL and RA collected the data. GL, RA, and EN-S performed the data analysis. GL wrote the first draft. All authors contributed to the conception and design of the study, contributed to the manuscript revision, read and approved the submitted version.

## Conflict of Interest

The authors declare that the research was conducted in the absence of any commercial or financial relationships that could be construed as a potential conflict of interest.
